# Cadmium Toxicity on Chondrocytes and the Palliative Effects of 1α, 25-Dihydroxy Vitamin D_3_ in White Leghorns Chicken's Embryo

**DOI:** 10.3389/fvets.2021.637369

**Published:** 2021-02-10

**Authors:** Jianhong Gu, Saihui Li, Guoshuai Wang, Xueqing Zhang, Yan Yuan, Xuezhong Liu, Jianchun Bian, Xishuai Tong, Zongping Liu

**Affiliations:** ^1^College of Veterinary Medicine, Yangzhou University, Yangzhou, China; ^2^Jiangsu Co-innovation Center for Prevention and Control of Important Animal Infectious Diseases and Zoonoses, Yangzhou, China; ^3^Jiangsu Key Laboratory of Zoonosis, Yangzhou, China; ^4^Institutes of Agricultural Science and Technology Development, Joint International Research Laboratory of Agriculture and Agri-Product Safety of the Ministry of Education of China, Yangzhou University, Yangzhou, China

**Keywords:** cadmium, 1α, 25-dihydroxyvitamin D3 [1α, 25-(OH)_2_D_3_], apoptosis, matrix metalloproteinase, chicken embryo chondrocytes

## Abstract

Cadmium (Cd) can causes osteoporosis and joint swelling. However, the mechanism of Cd toxicity in chondrocytes and how to alleviate Cd poisoning to chondrocytes are still unclear. Herein, we evaluated the toxicity of Cd to chicken chondrocytes, and whether vitamin D can relieve the toxicity of Cd to chondrocytes. Primary chondrocytes were collected from knee-joint cartilage of 15-day-old chicken embryos. They were treated with (0, 1, 2, and 4) μM Cd alone, 10^−8^ M 1α,25-(OH)_2_D_3_ alone, or 2 μM Cd combined with 10^−8^ M 1α,25-(OH)_2_D_3_. We found that Cd significantly inhibited *Sox9* and *ACAN* mRNA expression, which are markers for chondrocyte differentiation, downregulated the mitochondrial membrane potential, upregulated the Bax/B-cell lymphoma 2 ratio. Furthermore, Cd significantly promoted matrix metalloproteinase (MMP)-9 expression, thus accelerating the degradation of extracellular matrix. And Cd also inhibited the expression of main macromolecular protein of extracellular matrix, Collagen type IIα1 (COL2A1) and acid mucopolysaccharide. However, 1α,25-(OH)_2_D_3_ pretreatment significantly alleviated the toxicity effects of Cd on the differentiation, apoptosis and extracellular matrix gene expression in primary chondrocytes. Conclusively, Cd exposure could inhibited chicken embryo chondrocytes differentiation, extracellular matrix gene expression, and induced chondrocyte apoptosis. However, these toxic effects of Cd are alleviated by the pretreatment of chondrocytes with 1α,25-(OH)_2_D_3_.

## Introduction

Osteoarthritis (OA) is a chronic degenerative osteoarthropathy, which is characterized by the degeneration and the matrix degradation of cartilage (1). OA is not only a bone disease that has plagued human beings for a long time, but also a common disease in poultry. The occurrence of OA will cause the poultry to limp, ache, depression, decreased laying rate, and disease spread will cause serious economic losses to the farm. Li et al. (2) found that the concentrations of Cd in pig, dairy cow and chicken manures were positively correlated to those in their feeds (2). Feed is one of the main sources of cadmium in livestock, and cereal is the most significant contributor to the dietary intake of Cd (3). It has been confirmed that Cd can cause kidney and liver damage in poultry (4–7). Recently, Shen et al. (8) found that the deposition of Cd in cartilage had been overlooked in the past (8). This finding suggests that cadmium can directly cause cartilage damage.

Cadmium (Cd) mainly causes nephrotoxicity and osteotoxicity (9–13). Existing research on the Cd osteotoxicity at the cellular level is mostly focused on osteoclasts and osteoblasts (14, 15). Cd induces apoptosis in osteoblasts through mitochondrial damage and oxidative stress, reduces the secretion of collagen and other components of bone matrix as well as alkaline phosphatase (ALP) activity, and inhibits the differentiation of rat osteoblast through the Wnt/beta-catenin signaling pathway (16, 17). Low concentrations of Cd (7.5–60 nmol/L) also induce mice osteoclast differentiation and bone resorption through Ca^2+^/CaM/CaMK signaling (18). Antagonists of Cd mainly include zinc, selenium, and vitamins, which reduce the free Cd concentration in the body by inducing the synthesis of metallothionein or directly combining with Cd. Cd antagonists also reduce Cd-induced oxidative damage in the reproductive system, the respiratory system, the cardiovascular system, and the immune system (19–22).

Chondrocytes are originally derived from the bone marrow mesenchymal cells (BMSCs), which differentiate under the action of the transcription factor sex determining region Y box 9 (Sox9) during the early stage of differentiation (23). Chondrocytes are responsible for the production, maintenance, and degradation of collagen type IIα1 (COL2A1), proteoglycans, sulfated glycosaminoglycans, collagen fibers, and other macromolecules, which are important indicators for evaluating the physiological functions of chondrocytes. Matrix metalloproteinase-9 (MMP-9), belongs to the family of MMPs and is highly expressed in the late stage of chondrocyte differentiation to hypertrophy. MMP-9 degrades types I, II, and X collagens in the extracellular matrix, the core protein of proteoglycans, gelatin, fibronectin, and elastin and may even trigger apoptosis (24–26).

1α,25-dihydroxyvitamin D_3_ [1α,25-(OH)_2_D_3_] binds to the vitamin D receptor of epithelial cells in the small intestine to mediate calcium absorption and also directly acts on osteoblasts, osteoclasts, and chondrocytes to regulate proliferation and differentiation, directly participating in the processes of bone formation and bone resorption (27–30). It has been confirmed that the addition of 5–10 μg/kg 1α,25-(OH)_2_D_3_ to broiler feed greatly reduces the occurrence of cartilage dysplasia in the animals and also improves the activity of chondrocytes in the inflammatory state, inhibits the degradation of the cartilage extracellular matrix COL2A1 and ACAN, and protects the articular cartilage (31, 32). A study by El-Boshy et al. (33) showed that vitamin D and calcium enhanced the antioxidant and anti-inflammatory effects and reduce Cd-induced rat hepatotoxicity (33). However, whether 1α,25-(OH)_2_D_3_ reduces the toxic effect of Cd on chondrocytes and, if so, how 1α,25-(OH)_2_D_3_ reduces these effects remains unclear. This study evaluated the effects of Cd and 1α,25-(OH)_2_D_3_ co-treatment on the differentiation, apoptosis, and main components of the extracellular matrix of primary culture chicken embryo chondrocytes and examined whether 1α,25-(OH)_2_D_3_ reduced the toxic damage to chicken primary chondrocytes caused by Cd.

## Materials and Methods

### Experimental Animals

The chicken embryos we use are fertilized specific-pathogen-free (SPF) grade eggs from Single Comb White Leghorn (Yigida Biotechnology, China). SPF grade eggs were incubated at 37°C with 60% humidity and incubated from E1 (1-day-old embryos). This study strictly followed the recommendations of the *Guidelines for the Care and Use of Laboratory Animals* issued by the National Research Council. The chicken embryos were euthanized by cervical dislocation. All animal experiments and procedures were approved by the Animal Care and Use Committee of Yangzhou University, China [Approval number: SYXK (Su) 2016-0020].

### Isolation and Culture of Chicken Primary Chondrocytes

Chicken embryos were isolated according to the previously published studies (34, 35). After discarding the supernatant, the pellet was washed with serum-free DMEM (Gibco, USA) and once with DMEM containing 10% fetal bovine serum (FBS, Eallbio, USA). A 4 ml volume of DMEM containing 10% FBS was then added and a pipette tip was used to gently resuspend the pellet. The collected cell suspension was filtered with a 400-mesh filter and the cells were counted. The cells were seeded in a 6-well plate (5.0 × 10^5^/well) and cultured in a 37°C constant temperature incubator with a volume fraction of 5% CO_2._ The above procedures strictly followed aseptic protocols, and the culture medium was changed every 48 h.

### 1α,25-(OH)_2_D_3_ and Cadmium Acetate Treatment in Chondrocytes

The experiment was actually carried out in four stages. In the first stage, we investigated the phenotypic changes and differentiation of chicken embryo primary chondrocytes during *in vitro* culture. The chondrocytes proliferated slowly during the first 3 days of *in vitro* culture. Chondrocytes showed hypertrophy and characteristic chondrocyte differentiation phenotype on the 5th day. On the 7th day of prolonged culture, the characteristics of chondrocyte hypertrophy became more obvious. Therefore, in the second stage, we selected 3, 5, and 7 d time points to detect the expression of genes and proteins related to chondrocytes early differentiation.

In the second stage, we only investigated the effects of 10^−8^ M 1α,25-(OH)_2_D_3_ on chicken embryo primary chondrocytes. The experimental group was added with 10^−8^ M 1α,25-(OH)_2_D_3_, and the control group was not added with 10^−8^ M 1α,25-(OH)_2_D_3_. The chicken embryo primary chondrocytes were cultured *in vitro* until the 7th day, and the expression of genes and proteins related to early differentiation in the control group and the experimental group were detected at the time points of 3, 5 and 7 days.

In the third stage, we only investigated the effects of different concentrations of Cd on chicken embryo primary chondrocytes. We selected multiple concentrations of Cd first in order to determine an appropriate concentration of Cd for the fourth stage. We first selected seven concentrations (0, 0.5, 1, 2, 4, 6, 8, or 10 μM) of Cd to treat chondrocytes and detected the apoptosis level. It was found that Cd above 2 μM significantly promoted the apoptosis of chondrocytes. Then we selected four concentrations (0, 1, 2, 4 μM) of Cd to treat chondrocytes and detected the expression of early differentiation genes and extracellular matrix proteins of chondrocytes, and it was found that 2 μM Cd significantly decreased the expression of Sox9 and COL2A1. Due to the extremely significant changes caused by 2 μM Cd in chondrocytes, we decided to use 2 μM Cd in the fourth stage.

In the fourth stage, we selected a combination of 10^−8^ M 1α,25-(OH)_2_D_3_ and 2 μM Cd to treat chicken embryo primary chondrocytes, and observe whether 1α,25-(OH)_2_D_3_ can reduced the toxic damage to chicken primary chondrocytes caused by Cd. We are divided into four groups, please find the Table 1.

**Table 1:** The grouping of Cd and 1α,25-(OH)_2_D_3_ combined treatment.

**Groups**	**con**	**1α,25-(OH)_**2**_D_**3**_**	**Cd**	**1α,25-(OH)_**2**_D_**3**_+Cd**
2 μM Cd	-	-	+	+
10^−8^ M 1α,25-(OH)_2_D_3_	-	+	-	+

### Alcian Blue Staining

The Alcian blue staining kit (Sigma, USA) was used to identify the chondrocytes and analyze the secretion of acid mucopolysaccharide from chondrocytes in this study after referring to the experimental method of Ovchinnikov (36). After taking the culture plate out of the incubator and discarding the medium, the chondrocytes were rinsed 3 times with PBS for 5 min each time, then fixed in 4% paraformaldehyde at room temperature for 30 min and rinsed 3 times with PBS. After discarding the PBS, the chondrocytes were maintained with 0.1 mol/L HCl for 5 min to lower the pH to 1.0, and then stained in 1% Alcian blue dye solution overnight at room temperature. The chondrocytes were then rinsed with 0.1 mol/L HCl to remove the background stain, photographed under a light microscope at 200 × (Leica Germany), and the ratio of the alcian blue-stained area to the total cell area was measured using Image Pro software (Media Cybernetics).

### Immunofluorescence Staining of Type II Collagen

The chondrocytes were inoculated in a 12-well plate (cell density: 2.0 × 10^5^/well), and then treated with 10^−8^ M 1α,25-(OH)_2_D_3_ and/or 2 μM Cd acetate for 72 h. After treatment, the chondrocytes were washed in PBS for 2 min on a shaker, fixed in 4% paraformaldehyde at room temperature for 30 min and subsequently washed in PBS 3 times. The chondrocytes were then treated with 0.4% Triton X-100 (Amresco, USA) for 20 min, the antigens blocked with 5% bovine serum albumin at room temperature for 30 min, incubated with anti-COL2A1 primary antibodies (Abcam, UK) overnight at 4°C, washed 3 times in PBS, and then incubated with fluorescence secondary antibodies (Cell Signaling, USA) in the dark for 1 h. The cytoskeletons of chondrocytes were stained with phalloidin solution at room temperature for 15 min. After rinsing with PBS, the chondrocytes were further incubated with 4',6-diamidino-2-phenylindole (DAPI) solution (Beyotime, China) at room temperature for 5 min nuclear staining. The glass slip in each culture plate well was removed and placed upside down on a glass slide with anti-fluorescence quenching and mounting medium, sealed with nail polish, air-dried, and stored in the dark at 4°C before observing and photographing under a laser confocal microscope (Leica).

### RNA Eextraction and RT-qPCR

Total RNA was extracted from chondrocytes using TRIzol reagent (Life Technologies, USA) and RNA concentration was quantified using a NanoDrop 2000 spectrophotometer (Thermo fisher). The extracted RNA from different groups was subjected to reverse transcription using Prime Script^TM^ RT Reagent Kits (Takara Bio, Japan), according to the manufacturer's instructions, to obtain the corresponding cDNAs, which were stored at −70°C after dilution. The cDNA template of each group was used for RT-qPCR, which was done with Premix Ex Taq^TM^ Kits (Takara Bio, Japan), according the manufacturer's instructions, using an ABI7500 RT-qPCR system (ABI). The RT-qPCR conditions were 40 cycles of 95°C for 10 s and 60°C for 30 s. Glyceraldehyde 3-phosphate dehydrogenase (*GAPDH*) was used as the internal reference gene for the reaction. The relative expression of the target genes was calculated *via* the 2^−ΔΔCT^ method based on the results of the RT-qPCR. Table 2 shows the primer sequences used for RT-qPCR.

**Table 2:** Primer sequences of target genes.

**Target genes**	**Forward primer (5**^′^**-3^**′**^)**	**Reverse primer (5**^′^**-3^**′**^)**
*ACAN* (chicken)	TGGGCGTGCGGACCGTTTA	TGGGCTCCAGGGTAGCGATG
*Sox9*(chicken)	ACTGAGCGGTGAGCAGGGTC	AGGTGAAGGTGGAGTAGAGGC
*GAPDH* (chicken)	ATGGCATCCAAGGAGTGA	GGGAGACAGAAGGGAACAG

### Protein Extraction and Western Blot

The chondrocytes were lysed with radioimmunoprecipitation assay (RIPA) buffer (Beyotime, China), followed by ultrasonication and lysing to extract the total protein, which was quantified using a bicinchoninic acid assay (Beyotime, China). After adjusting the protein concentration to a consistent level, the diluted total protein was added to 6 × SDS-PAGE loading buffer (NCM Biotech, China) at a ratio of 1: 5, boiled for 10 min, and stored at −80°C for later use.

The same amount of total protein (20 μg) was separated in 8% and 12% SDS-PAGE gels based on the molecular weights of the target proteins, and the proteins were then transferred from the gel to polyvinylidene fluoride or polyvinylidene difluoride (PVDF) membranes (Merck Millipore). The PVDF membranes were blocked with tris-buffered saline and 0.1% Tween (TBST) solution containing 5% skim milk for 2 h and then by incubated with anti-COL2A1 (Sigma, USA), anti-MMP-9, anti-Bax, or anti-Bcl-2 primary antibodies (Cell Signaling, USA) at 4°C overnight. After washing the membranes in TBST 3 times, the membranes were incubated with the corresponding horseradish peroxidase (HRP)-conjugated goat anti-mouse and goat anti-rabbit secondary antibodies (Cell Signaling, USA) at room temperature for 2 h. Finally, the PVDF membranes were developed using enhanced chemiluminescence (ECL) substrate before the protein bands were displayed in an automated chemiluminescence imaging system (Tanon-5200). The gray values of the corresponding protein bands were analyzed using the Image J software (National Institutes of Health).

### Flow Cytometry

A fluorescein isothiocyanate (FITC) Annexin Apoptosis Detection Kit (Vazyme Biotech, USA) was used to detect the chondrocyte apoptosis. The chondrocyte culture was removed from the incubator and the culture medium and cells were collected in a flow cytometry tube, centrifuged at 1, 000 rpm for 10 min and the supernatant discarded. The cell pellet was then washed in twice in PBS and 300 μl 1 × Annexin V binding buffer was added to the pellet followed by FITC and propidium iodide dyes. The pellet was then incubated at 37°C in the dark for 30 min, buffer was then added to reach a final volume of 500 μl and chondrocyte apoptosis was detected *via* a flow cytometer (BD Biosciences).

A JC-1 mitochondrial membrane potential detection kit (Beyotime, China) was used to detect the mitochondrial membrane potential of chondrocytes. The chondrocyte culture was removed from the incubator, the culture medium and cells were collected in a flow cytometry tube, centrifuged at 1,000 rpm for 10 min and the supernatant discarded. The cell pellet was washed once in PBS and then incubated with the JC-1 staining and working solution at 37°C in the dark for 20 min. After incubation, the cells were washed twice with pre-cooled 1 × JC staining buffer and subsequently resuspended in an appropriate amount of buffer before the mitochondrial membrane potential was measured with a flow cytometer.

### Statistical Analysis

All data are presented as mean ± standard deviation. GraphPad Prism 6.0 software (GraphPad Software Inc.) was used for statistical analysis. One-way analysis of variance (ANOVA) was used to compare the mean among multiple groups. Figures showing all data and analyses were prepared using GraphPad Prism 6.0. *P*-values lower than 0.05 were condidered statistically significant.

## Results

### Identification of the Phenotype of Primary Culture Chicken Embryo Chondrocytes

**Figure 1:**
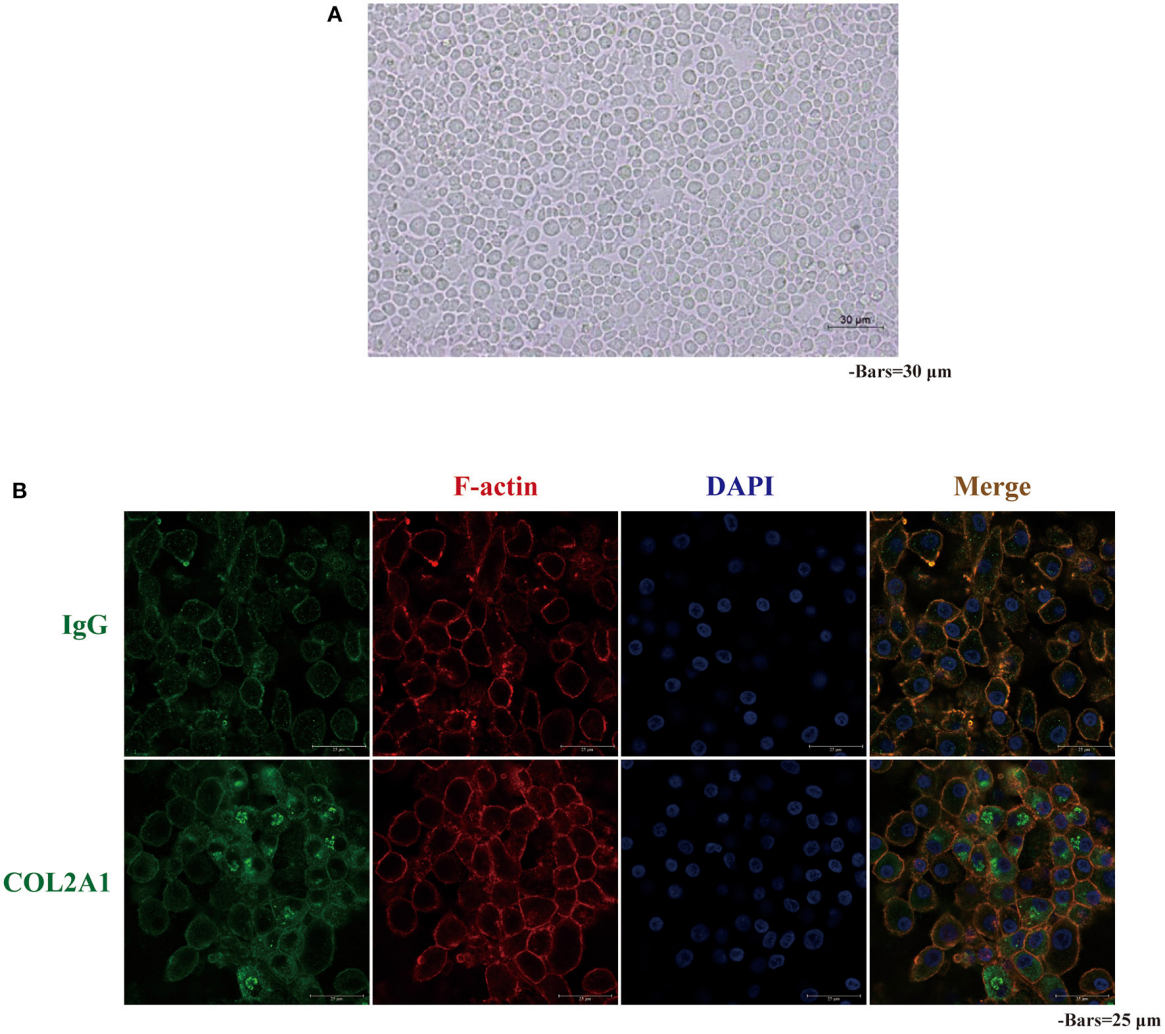
Morphology and COL2A1 expression in primary chicken chondrocyte cultures. **(A)** After 5 days of culture, the chicken primary chondrocytes were aggregated and had grown with a “paving stone” phenotype; **(B)** Representative images of immunofluorescence staining showing a strong green fluorescence signal in chicken primary chondrocytes expressing COL2A1. IgG was used as a negative control.

Stepwise digestion with trypsin and type IV collagenase was used to obtain articular chondrocytes from cartilage of chicken embryo knee-joints. The newly obtained chondrocytes were spherical, suspended, and relatively round. After culture in an incubator with 5% CO_2_ and constant temperature for ~2 h, some cells began to stick to the wall, and gradually became hypertrophic and tightly connected. The chondrocytes did not begin to show hypertrophy until day 5, and the local cells were adhered together and showed a typical paving stone-like morphology (Figure 1A). With the extension of the *in vitro* culture, the chondrocytes became more hypertrophic, and the paving stone-like morphology became more obvious, which is consistent with the phenotypic characteristics of normal chondrocyte differentiation. The COL2A1 immunofluorescence method was used to identify the obtained chondrocytes. As shown in Figure 1B, region positively stained for COL2A1 emitted strong green fluorescence and the immunoglobin (Ig)G negative control showed no green fluorescence, indicating that the obtained cells were COL2A1-positive chondrocytes.

### 1α,25-(OH)_2_D_3_ Maintained the Normal Differentiation Phenotype of Primary Chondrocytes and Promoted COL2A1 Expression in Chondrocytes

**Figure 2:**
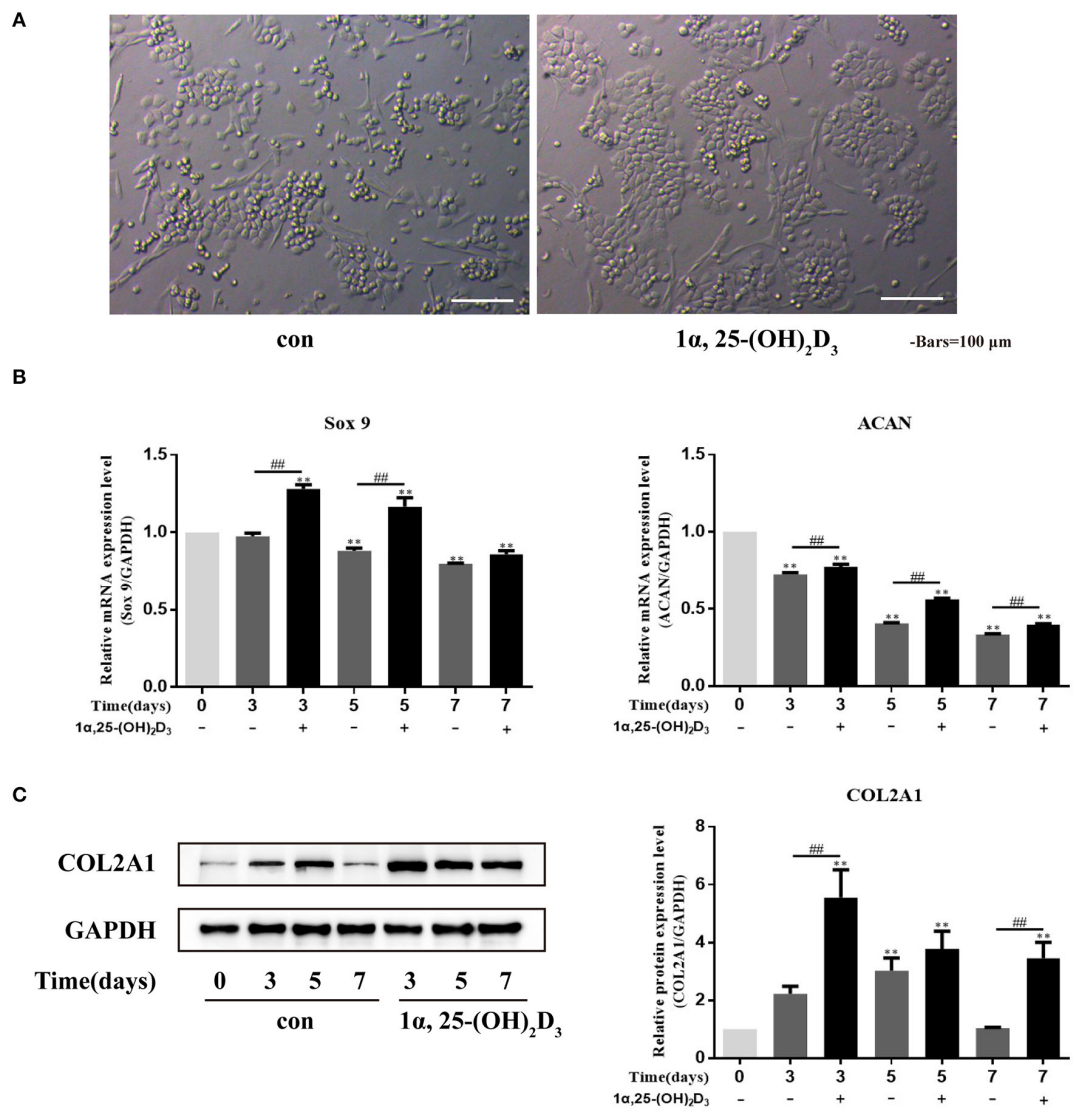
1α,25-(OH)_2_D_3_ promoted the differentiation of primary chondrocytes and the expression of COL2A1 protein. **(A)** 1α,25-(OH)_2_D_3_ maintained the “paving stone-like phenotype” of chondrocyte culture *in vitro*; **(B)** Relative expression of *Sox9* and *ACAN* mRNA, detected by RT-qPCR, in chondrocytes with or without the 0, 3, 5, or 7 days treatment with 10^−8^ M 1α,25-(OH)_2_D_3_ showed that 1α,25-(OH)_2_D_3_ upregulated the expression of *Sox9* and *ACAN* mRNA in chondrocytes; **(C)** Expression of COL2A1 protein, detected by Western blot, in chondrocytes with or without 0, 3, 5, or 7 days treatment with 10^−8^ M 1α,25-(OH)_2_D_3_ showed that 1α,25-(OH)_2_D_3_ upregulated the expression of COL2A1 protein. All data are presented as mean ± standard deviation; n = 3; *P < 0.05 and **P < 0.01, significant difference relative to the day 0 group; ^##^*P* < 0.01, significant difference between 1α,25-(OH)_2_D_3_ treatment and 1α,25-(OH)_2_D_3_ untreated groups at each time point.

Chondrocytes are prone to dedifferentiation during *in vitro* culture. To observe the effect of 1α,25-(OH)_2_D_3_ on the phenotype of chondrocytes *in vitro*, 10^−8^ M 1α,25-(OH)_2_D_3_ was added into the culture medium, and the control group was not added with 10^−8^ M 1α,25-(OH)_2_D_3_. Observation of the phenotypic changes of chondrocytes (Figure 2A) showed that, although the local cells in the control group appeared to have “paving stone-like,” long strips, or spindle-shaped phenotypes were seen in the control group. There was a significant reduction of long strips or spindle-shaped phenotypes observed in the 1α,25-(OH)_2_D_3_ treatment group. Most of the cells in the treatment group were in round or polygonal in shape, with a strong sense of three-dimensionality, and the local cells were obviously clustered, indicating that 1α,25-(OH)_2_D_3_ reduced the phenomenon of dedifferentiation and chaotic differentiation in chondrocytes and helped maintain the normal differentiation phenotype of primary chondrocytes.

*Sox9* and *ACAN* are important transcription factors that regulate the early differentiation of chondrocytes. To study the effect of 1α,25-(OH)_2_D_3_ on chondrocyte differentiation, the primary chondrocytes were treated with 10^−8^ M 1α,25-(OH)_2_D_3_ for 3, 5, or 7 days *in vitro*, and *Sox9* and *ACAN* gene expression were then detected by real-time quantitative PCR (RT-qPCR) (Figure 2B). *Sox9* and *ACAN* mRNA expression on days 5 and 7 was downregulated compared with the day 0 group. 1α,25-(OH)_2_D_3_ upregulated the expression of *Sox9* and *ACAN* mRNA compared with the untreated group. On days 3 and 5, the levels of *Sox9* mRNA in the 1α,25-(OH)_2_D_3_-treated group were significantly higher than that in the untreated group (*P* < 0.01). On days 3, 5, and 7, the levels of *ACAN* mRNA in the 1α,25-(OH)_2_D_3_-treated group were significantly higher than that in the untreated group (*P* < 0.01). These results indicate that 1α,25-(OH)_2_D_3_ promoted the expression of *Sox9* and *ACAN* genes related to early differentiation of chondrocytes and promoted early differentiation of the primary culture of chondrocytes of chicken embryos *in vitro*.

Western blot to detect COL2A1 expression after chondrocytes were treated with 10^−8^ M 1α,25-(OH)_2_D_3_ for 3, 5, or 7 days (Figure 2C). The expression of COL2A1 in untreated chondrocytes reached a peak on day 5 (*P* < 0.01) and subsequently declined. However, the expression of COL2A1 in the chondrocytes treated with 1α,25-(OH)_2_D_3_ reached a peak on day 3 (*P* < 0.01) and then subsequently declined. At the same time, the expression of COL2A1 was significantly upregulated on days 3 and 7 in the treated group compared to the untreated group (*P* < 0.01) suggesting that 1α,25-(OH)_2_D_3_ promotes the expression of COL2A1 in chicken primary chondrocytes.

### Cadmium Induced Apoptosis, Inhibited Early Differentiation, and Promoted MMP-9 Expression in Chondrocytes

**Figure 3:**
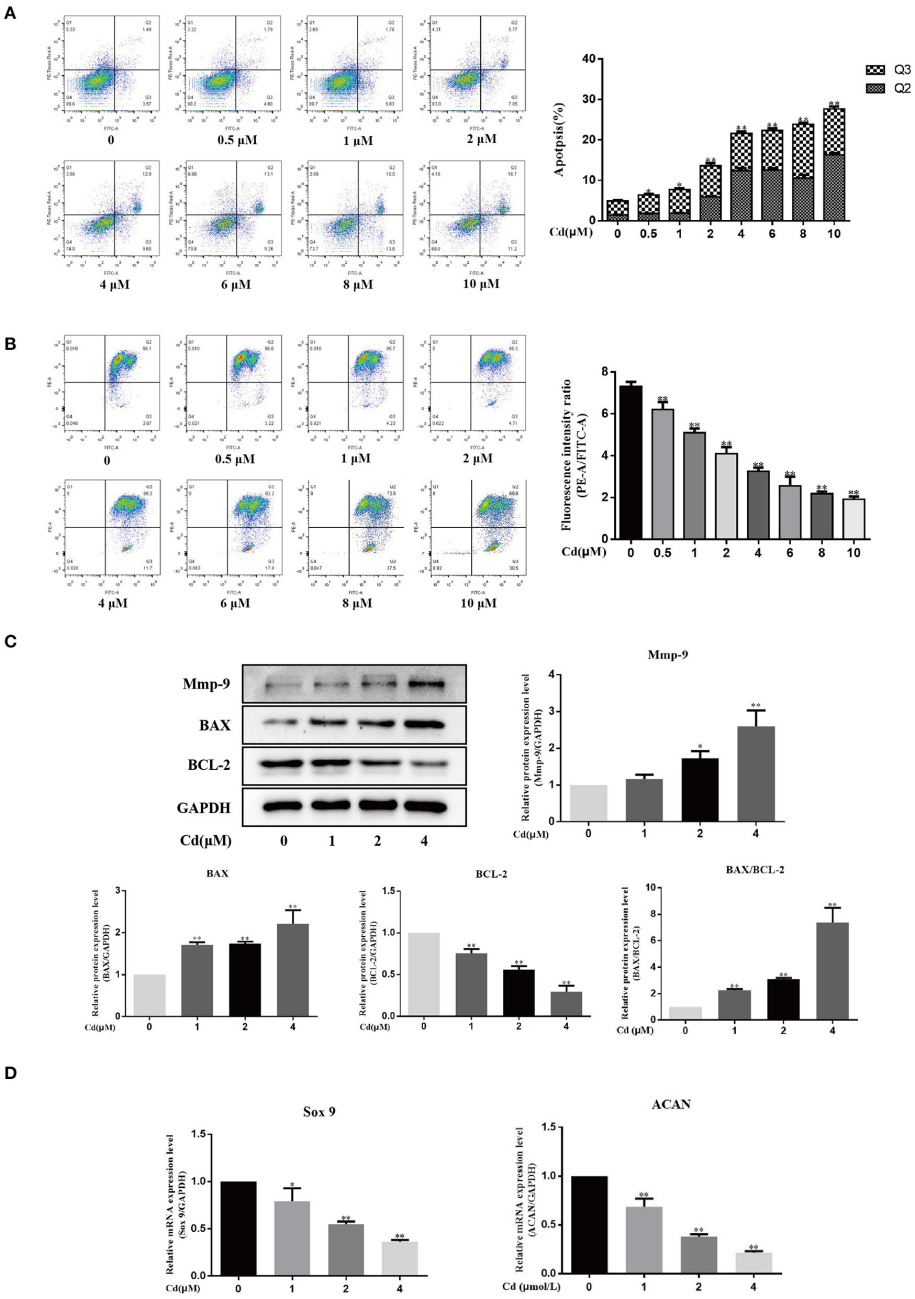
Cd inhibited chondrocyte differentiation and induced apoptosis and MMP-9 expression in chondrocytes. **(A)** Apoptosis rate of chondrocytes treated with different concentrations of Cd for 24 h. For flow cytometry, the Q1 quadrant represents the mechanical damage in chondrocytes, the Q2 quadrant represents late apoptosis in chondrocytes, the Q3 quadrant represents early apoptosis in chondrocytes, and the Q4 quadrant represents the normal chondrocytes. Compared with the control group, > 0.5 μM Cd induced apoptosis in the chondrocytes. **(B)** Changes in mitochondrial membrane potential in chondrocytes treated with different concentrations of Cd for 24 h. Compared with the control group, > 0.5 μM Cd significantly reduced the mitochondrial membrane potential in the chondrocytes. **(C)** Relative expression of MMP-9, Bax, and Bcl-2 in chondrocytes as detected by Western blot. Cd treatment upregulated MMP-9 and Bax protein expression, downregulated Bcl-2 protein expression, and increased the Bax/Bcl-2 ratio. **(D)** Expression of early differentiation markers, *Sox9* and *ACAN* mRNA as detected by RT-qPCR. Compared with the control group, Cd at concentrations above 1 μM inhibited the expression of *Sox9* and *ACAN* mRNA expression. All data are represented as mean ± standard deviation; n = 3; *P < 0.05 and **P < 0.01, significant difference relative to the day 0 group.

To evaluate the effect of Cd on the apoptosis of primary chondrocytes *in vitro*, the primary chondrocytes were treated with different concentrations of Cd (0, 0.5, 1, 2, 4, 6, 8, or 10 μM) for 24 h, and flow cytometry was then used to detect the apoptosis level (Figure 3A). Compared with the control group, > 0.5 μM Cd significantly induced the apoptosis of primary chondrocytes (*P* < 0.05), and >2 μM Cd significantly induced apoptosis in the primary chondrocytes (*P* < 0.01). Flow cytometry was also used to detect changes in mitochondrial membrane potential (Figure 3B). Compared with the control group, > 0.5 μM Cd effectively and significantly reduced the mitochondrial membrane potential of primary chondrocytes (*P* < 0.01), indicating that Cd induces apoptosis of primary chondrocytes *in vitro*.

In this study, Western blot was used to detect the expression of pro-apoptotic protein (Bax) and B cell lymphoma 2 (Bcl-2) in chondrocytes treated with Cd for 3 days (Figure 3C). Compared with the control group, the expression of Bax was significantly (*P* < 0.01) increased and the expression of Bcl-2 was significantly reduced (*P* < 0.01) with the increase in Cd concentration. Thus, the Bax/Bcl-2 ratio increased. These findings indicate that Cd promoted apoptosis in chondrocytes and are consistent with the flow cytometry results.

To study the effect of Cd on the early differentiation of chondrocytes, 0, 1, 2, or 4 μM Cd was used to treat the chondrocytes for 24 h and RT-qPCR was then used to detect *Sox9* and *ACAN* gene expression (Figure 3D). Compared with the control group, *Sox9* expression was significantly downregulated in chondrocytes treated with 1 μM Cd (*P* < 0.05) and was significantly downregulated in chondrocytes treated with >2 μM Cd (*P* < 0.01); *ACAN* expression was significantly downregulated in chondrocytes treated with >1 μM Cd (*P* < 0.01). These results indicate that Cd inhibits the early differentiation of chondrocytes.

To understand the effect of Cd on the expression of the extracellular matrix degradation protein, MMP-9, in chondrocytes, Western blot was used to detect MMP-9 expression in chondrocytes treated with Cd for 3 days (Figure 3C). Compared with the control group, 2 μM Cd significantly promoted the expression of MMP-9 (*P* < 0.05), and 4 μM Cd significantly promoted the expression of MMP-9 (*P* < 0.01), suggesting that Cd upregulated the expression of MMP-9 and inhibit the expression of chondrocyte extracellular matrix protein.

### Cadmium Inhibited the Expression of COL2A1 and Acid Mucopolysaccharide in Chondrocytes

Collagen and acid mucopolysaccharides are both important components of the chondrocyte extracellular matrix. Protein macromolecules, such as chondroitin sulfate, hyaluronic acid, glycosaminoglycans (GAGs), and ACAN are all acid mucopolysaccharides. Alcian blue specifically binds to acid mucosubstances; thus, alcian blue can be used to stain chondrocytes and the ratio of the alcian blue-stained chondrocyte area to the total cell area can be used to analyze the effect of Cd on the expression of acid mucopolysaccharide in chondrocytes.

**Figure 4:**
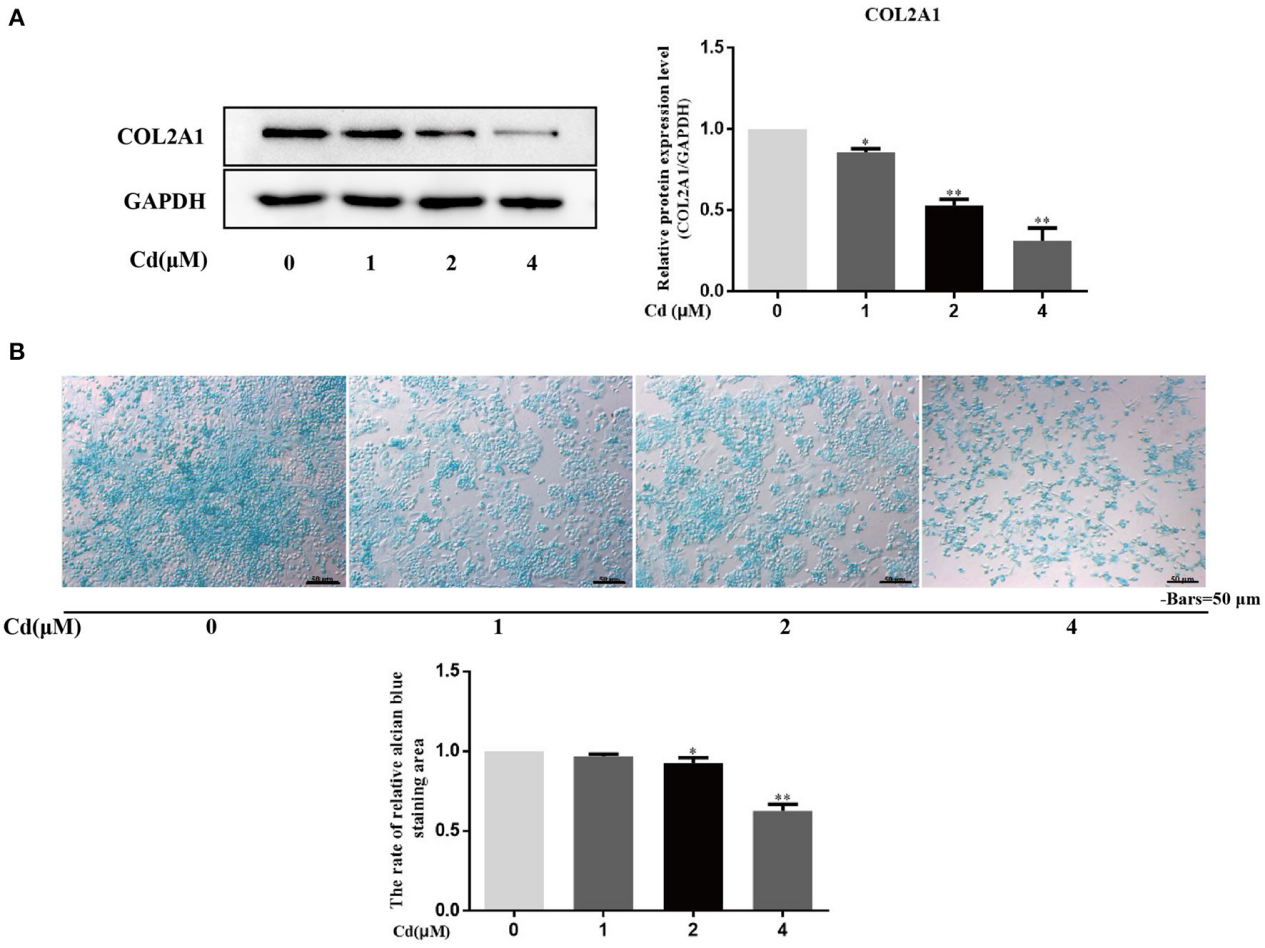
Cd inhibited the expression of COL2A1 and acid mucopolysaccharide in chondrocytes. **(A)** Western blot analysis detecting the relative expression of COL2A1 protein after chondrocytes were treated with Cd for 3 days showed that, compared with the control group, the expression of COL2A1 protein was reduced after >1 μM Cd treatment. **(B)** Alcian blue staining of primary chondrocytes treated with Cd for 3 days showed that >2 μM Cd significantly reduced the acid mucopolysaccharide in chondrocytes compared with the control group. All data are represented as mean ± standard deviation; n = 3; *P < 0.05 and **P < 0.01, significant difference relative to the day 0 group.

Primary chondrocytes were treated with 0, 1, 2, or 4 μM Cd for 3 days, and the expression of COL2A1 was detected in the different groups *via* Western blot (Figure 4A). Compared with the control group, treated with >1 μM Cd significantly reduced the expression of COL2A1 in the chondrocytes (*P* < 0.05), and treatment with >2 μM Cd significantly reduced the expression of COL2A1 in the chondrocytes (*P* < 0.01), suggesting that >1 μM Cd inhibited the expression of COL2A1 in the chondrocytes.

The effect of Cd on the secretion of acid mucopolysaccharides from chondrocytes was detected by alcian blue staining, and the ratio of the alcian blue-stained chondrocytes area to the total cell area was calculated (Figure 4B). Compared with the control group, no significant difference in the ratio of the alcian blue-stained chondrocyte area to the total cell area was found in the 1 μM Cd treatment group. Cd at concentrations above 2 μM significantly reduced the ratio of the alcian blue-stained chondrocyte area to the total cell area (*P* < 0.05), suggesting that Cd inhibited the secretion of acid mucopolysaccharide from chondrocytes.

### 1α,25-(OH)_2_D_3_ Inhibited the Effect of Cadmium on Apoptosis and MMP-9 Expression in Chondrocytes

**Figure 5:**
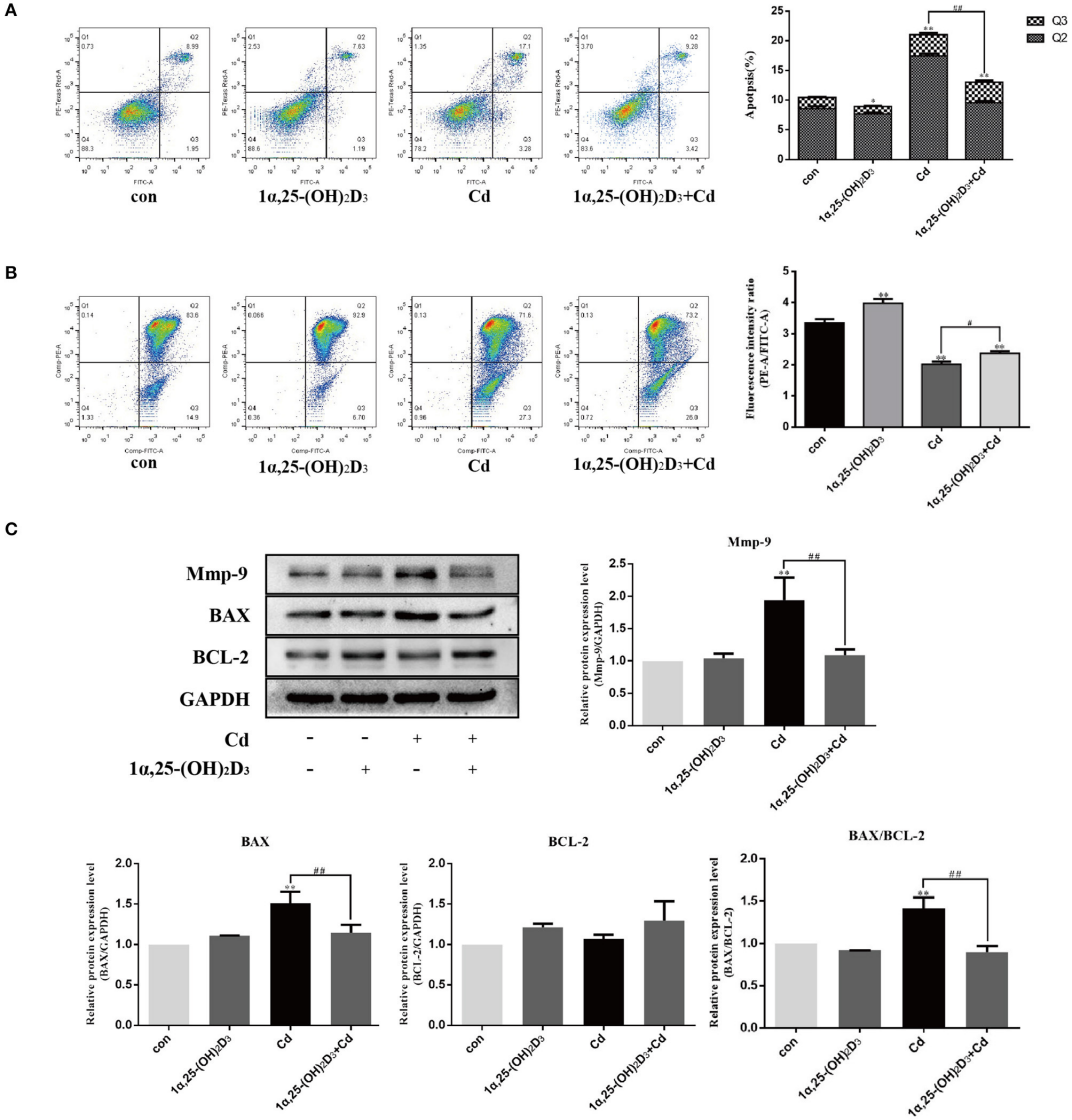
1α,25-(OH)_2_D_3_ inhibited the effect of Cd on apoptosis and MMP-9 expression in chondrocytes. **(A)** Apoptosis of chondrocytes after pretreatment with 10^−8^ M 1α,25-(OH)_2_D_3_ for 1 day followed by 1 day of combined treatment with 2 μM Cd. Compared with Cd treatment alone, the 1α,25-(OH)_2_D_3_ and Cd combination treatment reduced apoptosis in chondrocytes. **(B)** Mitochondrial membrane potential of chondrocytes pretreated with 10^−8^ M 1α,25-(OH)_2_D_3_ for 1 day followed by 1 day of combined treatment with 2 μM Cd. Compared with the Cd treatment alone, the 1α,25-(OH)_2_D_3_ and Cd combination treatment increased the mitochondrial membrane potential of the chondrocytes. **(C)** Expression of MMP-9, Bax, and Bcl-2 protein in chondrocytes pretreated with 10^−8^ M 1α,25-(OH)_2_D_3_ for 1 day followed by 3 days combined treatment with 2 μM Cd as detected by Western blot showed that, compared with the Cd treatment alone, the 1α,25-(OH)_2_D_3_ and Cd combination reduced Bax and MMP-9 expression, reduced the Bax/Bcl-2 ratio. All data are represented as mean ± standard deviation; n = 3; *P < 0.05 and **P < 0.01, significant difference relative to the day 0 group; ^#^*P* < 0.05 and ^##^*P* < 0.01, significant difference between the combined treatment group and Cd monotherapy group.

Previous studies have shown that Cd above 2 μM significantly induces apoptosis in chondrocytes. To further clarify whether 1α,25-(OH)_2_D_3_ had any influence on the pro-apoptotic effect of Cd, chondrocytes were treated with 2 μM Cd alone, 10^−8^ M 1α,25-(OH)_2_D_3_ alone, or Cd and 1α,25-(OH)_2_D_3_ combined for 24 h and the apoptotic rate of chondrocytes in different treatment groups was subsequently detected *via* flow cytometry (Figure 5A). Compared to treatment with Cd alone, treatment with 1α,25-(OH)_2_D_3_ and Cd combined significantly reduced the apoptotic rate of chondrocytes (*P* < 0.01), suggesting that 1α,25-(OH)_2_D_3_ inhibited the pro-apoptotic effect of Cd. Flow cytometry was used to detect changes in the mitochondrial membrane potential in primary chondrocytes (Figure 5B). Compared with Cd treatment alone, the combination of 1α,25-(OH)_2_D_3_ and Cd significantly increased the mitochondrial membrane potential of the chondrocytes (*P* < 0.05), suggesting that 1α,25-(OH)_2_D_3_ reduced the mitochondrial membrane potential in chondrocytes caused by Cd.

Western blot was used to detect the expression of the chondrocyte-related apoptosis proteins (Figure 5C). Compared with the control group, the Cd treatment alone significantly increased the expression of Bax in the chondrocytes (*P* < 0.01). Compared with Cd treatment alone, 1α,25-(OH)_2_D_3_ and Cd combined significantly reduced the expression of Bax in the chondrocytes (*P* < 0.01), but did not significantly affect the expression of Bcl-2. However, the Bax/Bcl-2 ratio decreased, suggesting that 1α,25-(OH)_2_D_3_ alleviated the pro-apoptotic effect of Cd on primary chondrocytes.

Western blot was used to detect the effect of 1α,25-(OH)_2_D_3_ and Cd on the expression of MMP-9 in chondrocytes (Figure 5C). Compared with the control group, Cd treatment alone significantly increased the expression of MMP-9 in chondrocytes (*P* < 0.01). Compared with Cd treatment alone, 1α,25-(OH)_2_D_3_ and Cd combined significantly reduced the expression of MMP-9 in chondrocytes (*P* < 0.01), indicating that 1α,25-(OH)_2_D_3_ inhibited the upregulation of MMP-9 in the chondrocytes due to Cd and alleviated the degradation of macromolecules in the cartilage matrix, thereby protecting the chondrocytes.

### 1α,25-(OH)_2_D_3_ Relieved the Inhibitory Effect of Cadmium on the Expression of COL2A1 and Acid Mucopolysaccharide in Chondrocytes

**Figure 6:**
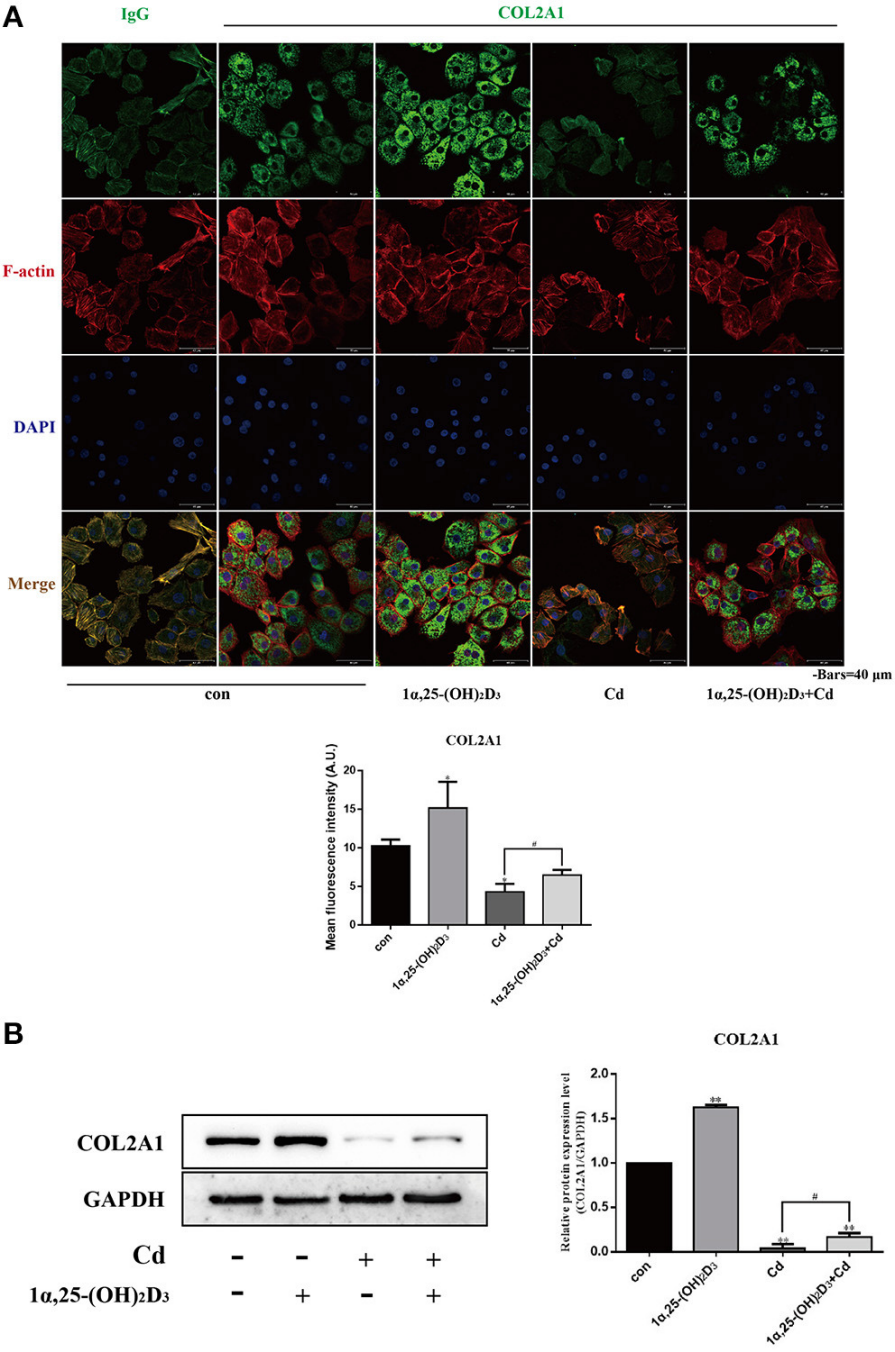
1α,25-(OH)_2_D_3_ relieved the inhibitory effect of Cd on the expression of COL2A1 in chondrocytes. **(A)** Immunofluorescence staining of COL2A1 in chondrocytes pretreated with 10^−8^ M 1α,25-(OH)_2_D_3_ for 1 day followed by 3 days of combined treatment with 2 μM Cd. Compared with Cd treatment alone, the 1α,25-(OH)_2_D_3_ and Cd combination treatment significantly increased the fluorescence signal of COL2A1 in chondrocytes. **(B)** Western blot of COL2A1 protein expression in chondrocytes pretreated for 1 day with 10^−8^ M 1α,25-(OH)_2_D_3_ followed by 3 days of combined treatment with 2 μM Cd for 3 days. Compared with the Cd treatment alone, the 1α,25-(OH)_2_D_3_ and Cd combination treatment significantly increased COL2A1 expression in chondrocytes. All data are represented as mean ± standard deviation; n = 3; *P < 0.05 and **P < 0.01, significant difference relative to the day 0 group; ^#^*P* < 0.05 and ^##^*P* < 0.01, significant difference between the combination treatment group and Cd monotherapy group.

The above findings show that Cd inhibited the expression of COL2A1 and acid mucopolysaccharides, while 1α,25-(OH)_2_D_3_ promoted the expression of COL2A1, suggesting that 1α,25-(OH)_2_D_3_ may alleviate the inhibitory effect of Cd on COL2A1 and even on acid mucopolysaccharides. Based on these results, primary chondrocytes were treated with 2 μM Cd alone, 10^−8^ M 1α,25-(OH)_2_D_3_ alone, or Cd combined with 1α,25-(OH)_2_D_3_ for 72 h and immunofluorescence staining was subsequently used to observe the effects of Cd and 1α,25-(OH)_2_D_3_ on the expression of COL2A1 in the chondrocytes (Figure 6A). The immunofluorescence intensity analysis results showed that the green fluorescence intensity was significantly increased in the 1α,25-(OH)_2_D_3_ treatment group (*P* < 0.05) and was significantly reduced in the Cd treatment group (*P* < 0.05) compared with the control group. Green fluorescence intensity with 1α,25-(OH)_2_D_3_ and Cd combined was significantly higher compared with the Cd treatment alone (*P* < 0.05), indicating that 1α,25-(OH)_2_D_3_ alleviated the inhibitory effect of Cd on the expression of COL2A1 in chondrocytes.

Results of Western blot (Figure 6B) showed that compared with the control group, the expression of COL2A1 in the chondrocytes was significantly increased in the 1α,25-(OH)_2_D_3_-treated group (*P* < 0.01) and significantly reduced in the Cd-treated and combined 1α,25-(OH)_2_D_3_ and Cd-treated groups (*P* < 0.01), indicating that 1α,25-(OH)_2_D_3_ promoted the expression of COL2A1, while Cd inhibited the expression of COL2A1 in the chondrocytes. Compared with the Cd treatment alone, 1α,25-(OH)_2_D_3_ and Cd combined significantly increased the expression of COL2A1 in chondrocytes (*P* < 0.01), which is consistent with the results of immunofluorescence staining and further demonstrates that 1α,25-(OH)_2_D_3_ alleviated the inhibitory effect of Cd on the expression of COL2A1 in chondrocytes.

**Figure 7:**
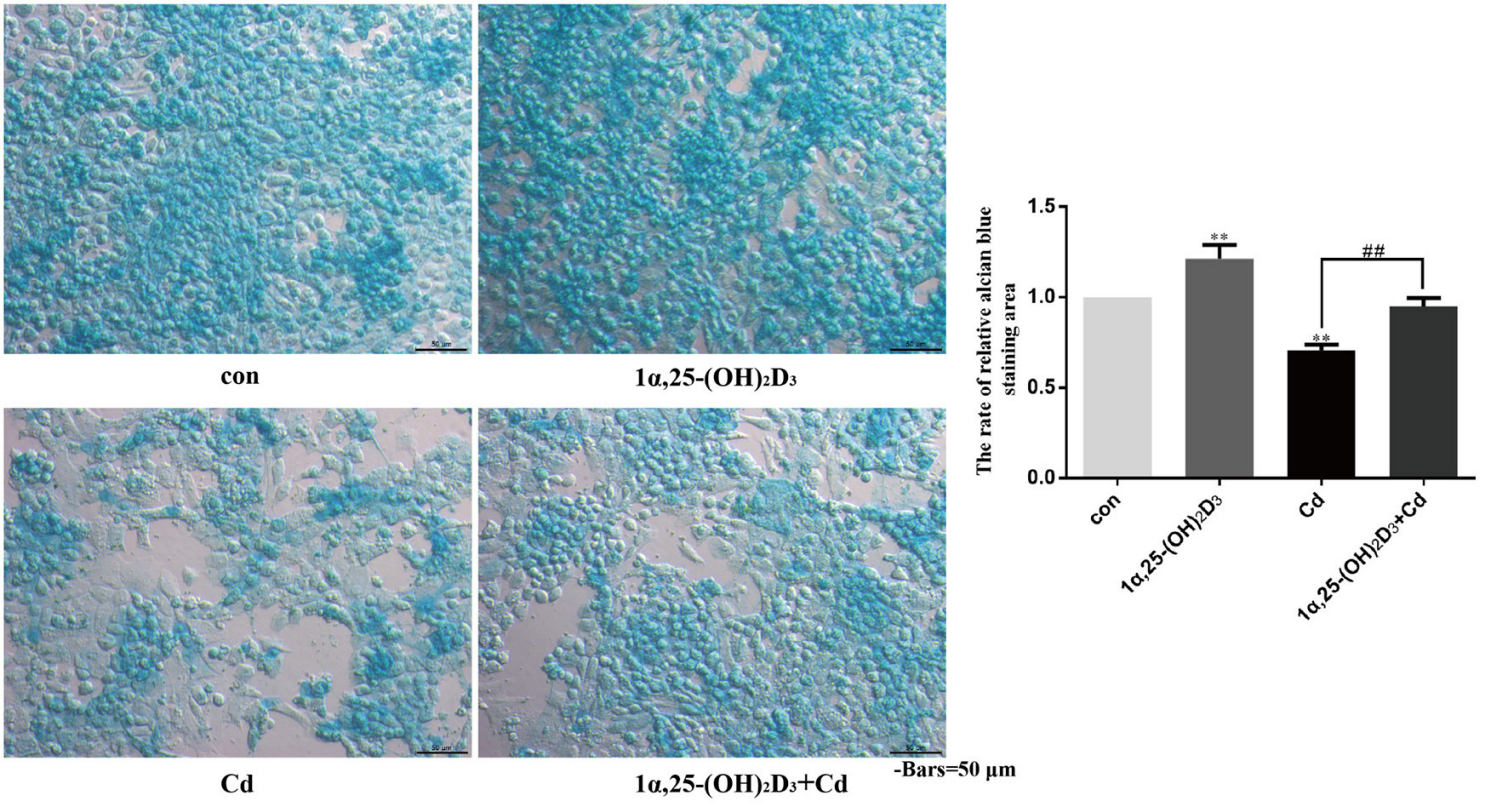
1α,25-(OH)_2_D_3_ alleviated the inhibitory effect of Cd on the expression of acid mucopolysaccharide in chondrocytes. Alcian blue staining was used to detect the acid mucopolysaccharide in chondrocytes pretreated with 10^−8^ M 1α,25-(OH)_2_D_3_ for 1 day followed by 3 days of combined treatment with 2 μM Cd. Compared with the control group, 1α,25-(OH)_2_D_3_ treatment alone increased the proportion of the alcian blue-stained area to the total cell area and Cd treatment alone reduced the proportion of the alcian blue-stained area to the total cell area. Compared with Cd treatment alone, the 1α,25-(OH)_2_D_3_ and Cd combination treatment increased the proportion of the alcian blue-stained area to the total cell area. All data are represented as mean ± standard deviation; n = 3; *P < 0.05 and **P < 0.01, significant difference relative to the day 0 group; ^#^*P* < 0.05 and ^##^*P* < 0.01, significant difference between the combination treatment group and Cd monotherapy group.

Chondrocytes were treated with 2 μM Cd alone, 10^−8^ M 1α,25-(OH)_2_D_3_ alone, or 1α,25-(OH)_2_D_3_ and Cd combined for 72 h and then stained with alcian blue to observe the effects of Cd and 1α,25-(OH)_2_D_3_ on acid mucopolysaccharide expression (Figure 7). Compared with the control group, 1α,25-(OH)_2_D_3_ treatment alone significantly increased the percentage of the alcian blue-stained area (*P* < 0.01), and Cd treatment alone significantly reduced the percentage of the alcian blue-stained area (*P* < 0.01), indicating that 1α,25-(OH)_2_D_3_ promoted the expression of acid mucopolysaccharide in chondrocytes, while Cd inhibited the expression of acid mucopolysaccharide in chondrocytes. Compared with the Cd treatment alone, the 1α,25-(OH)_2_D_3_ and Cd combined treatment significantly increased the proportion of the alcian blue-stained area in the chondrocytes (*P* < 0.01), indicating that 1α,25-(OH)_2_D_3_ alleviated the Cd inhibition of acid mucopolysaccharide expression in chondrocytes.

## Discussion

Cadmium is a serious environmental heavy metal pollutant, which has a half-life of 10–30 years, is widely deposited in the stomachs, intestines, livers, pancreases, testes, ovaries, bone marrow, lungs, muscles and fats of humans and animals, and induces apoptosis and autophagy (37, 38). Studies on Cd toxicity in bone tissue have shown that Cd turns mesenchymal stem cells from osteogenic differentiation to adipogenic differentiation, and promotes the generation of osteoclasts with bone resorption, thereby affecting bone homeostasis and reducing long bone density and calcium content (39–41). Kalisińska et al. showed that the content of Cd in cartilage tissue is higher than in bones and interferes with the absorption of copper, zinc, iron, manganese, and other trace elements in cartilage, thus causing osteoarthritis and osteoporosis (42, 43). Given that vitamin D enhances the antioxidant and anti-inflammatory effects and reduces the hepatotoxicity associated with Cd (33), we speculate that 1α,25-(OH)_2_D_3_ may also reduce the toxic damage to chondrocytes caused by Cd. Thus, we explored the effects of the interaction between 1α,25-(OH)_2_D_3_ and Cd and found that 1α,25-(OH)_2_D_3_ alleviated the toxic damage from Cd on the differentiation, apoptosis and expression of extracellular matrix protein COL2A1 and acid mucopolysaccharide in chicken primary chondrocytes.

Articular cartilage is composed of water, chondrocytes, COL2A1, and ACAN-based proteoglycans. The structure and composition of articular cartilage are affected by age, sex, genes, and the external environment. COL2A1, and ACAN provide the basis that enables the cartilage matrix to maintain stable metabolism and normal physiological and biochemical functions. COL2A1 is mainly produced by chondrocytes. It is the main protein macromolecule in the cartilage extracellular matrix, ensures the tightness and biomechanical properties of cartilage (44), and is also a marker for proliferative chondrocytes. The main proteoglycan in cartilage is the ACAN macromolecular protein, which interacts with hyaluronic acid to form macromolecular polymers that stably aggregate in the cartilage matrix and replenish water to resist compression and provide good elasticity (45). Arthritis, articular cartilage damage and degradation are related to the early degradation of collagen and proteoglycans secreted by chondrocytes and the destruction of molecular structure (46). Therefore, these macromolecules are the main indicators for the functional identification of chondrocytes *in vitro* and the indicators for detecting cartilage damage. The main enzymes that degrade collagen are collagenases and MMPs. A variety of factors activate MMP-1, MMP-2, MMP-3, MMP-8, MMP-9, MMP-13, and collagenase through interleukin (IL)-1, IL-5, and other inflammatory cytokines to degrade the extracellular matrix of articular cartilage, leading to an imbalance of the cartilage environment (47). Under the joint action of multiple members of the MMP family, collagen fibrils are degraded, resulting in irreversible damage to articular cartilage. Thus, COL2A1 is an important therapeutic target for diseases with cartilage injury (48, 49). The normal proliferation and differentiation process of chondrocytes is inseparable from the regulation of transcription factors at the gene level. Chondrocytes at different stages of differentiation are regulated by different genes. Some researchers have shown that when *Sox9* mutations occur, the process of bone formation and the longitudinal extension of the cartilage growth plate are blocked (50). It has been found that *Sox9* is involved in the regulation of cartilage growth and development and is a marker gene for early differentiation in chondrocytes (51). *In vitro* culture of chondrocytes provided a good cell model for the study of the mechanisms involved in Cd-induced cartilage toxicity and Cd-induced cartilage toxicity overall in this study. The articular cartilages were separated from knee-joint of 15-day-old chicken embryos and primary chondrocytes were obtained by stepwise digestion with trypsin and type IV collagenase. The obtained chondrocytes expressed *Sox9, ACAN*, and marker proteins, such as COL2A1 and MMP-9, laying the foundation for the further experiments.

Similar to Wu et al. who reported that Cd inhibited the differentiation of bone marrow mesenchymal stem cells into osteoblasts, this study also found that Cd inhibited the expression of *Sox9, ACAN* mRNA and COL2A1 protein and the differentiation of chondrocytes (17). After 10^−8^ M 1α,25-(OH)_2_D_3_ treatment, the inhibitory effects of Cd on the proliferation of chondrocytes and the expression of *Sox9* and *ACAN* mRNA and COL2A1 protein were alleviated. The number of vitamin D receptors in the chondrocytes of tibial growth plates of poultry with tibial chondrocyte dysplasia was reduced, and chondrocyte differentiation and maturation were also blocked, confirming the important role of vitamin D in chondrocyte differentiation (52), and indicating that 1α,25-(OH)_2_D_3_ alleviated the inhibitory effect of Cd on chondrocyte differentiation.

Cd not only inhibits cell differentiation, but also upregulates the expression of the pro-apoptotic protein, Bax and downregulates the expression of the anti-apoptotic protein, Bcl-2, inducing oxidative stress and apoptosis in duck renal tubular epithelial cells through the mitochondrial pathway (53). In this study the effects of Cd on the expression of chondrocyte apoptosis-related proteins, Bax and Bcl-2, the apoptosis rate, and the mitochondrial membrane potential in chondrocytes was observed. The results showed that Cd upregulated the expression of Bax protein, inhibited the expression of Bcl-2 protein, significantly induced apoptosis and reduced mitochondrial membrane potential in chondrocytes. Pretreatment with 10^−8^ M 1α,25-(OH)_2_D_3_ alleviated the Cd-induced toxic effects on apoptosis and mitochondrial membrane potential in chondrocytes. This is consistent with a previous report that vitamin D deficiency causes oxidative stress in tissues and organs, damages mitochondria, and induces apoptosis (54), indicating that vitamin D alleviated the toxic damage to chondrocyte mitochondria and inhibited the apoptosis in chondrocytes caused by Cd.

Cartilage extracellular matrix is a dynamic network structure that exists between cells and is mainly composed of type II collagen, proteoglycan, and hyaluronic acid. MMPs are proteolytic enzymes that use extracellular matrix components as hydrolyzed substrates. They affect the dynamic balance of degradation and recombination by hydrolyzing extracellular matrix components and by participating in a variety of physiological and pathological processes in cells. MMP-9 positive cells are found in the mandibular condylar and limb bud cartilage of 14-day-old fetal mice. In addition, MMP-9 is very important in endochondral ossification, homeostasis of the extracellular matrix, and reconstruction of the joint disc. MMP-9 is overexpressed in the synovial tissue after temporomandibular joint injury (55, 56). The current study showed that Cd inhibited the expression of macromolecules, COL2A1 and acid mucopolysaccharides in the extracellular matrix of cartilage, and promoted MMP-9 protein expression. This is very similar to results of a previous report in which repeated exposure to Cd atomization was found to induce the expression of MMP-9 in rat lung tissues suggesting that Cd accelerates the degradation of cartilage extracellular matrix components *via* MMPs (57). In the current study, 1α,25-(OH)_2_D_3_ alleviated the inhibitory effect of Cd on the expression of COL2A1 and acid mucopolysaccharide in chondrocytes and reduced the high expression of MMP-9 induced by Cd. These results are consistent with a previous report in which 1α,25-(OH)_2_D_3_ inhibited the expression of MMP-9 in the lung tissues of patients with tuberculosis (58). These findings indicate that 1α,25-(OH)_2_D_3_ inhibits the expression of MMP-9, thus alleviating the Cd-induced toxic damage to the extracellular matrix. Exploring the interaction between 1α,25-(OH)_2_D_3_ and Cd on chondrocyte differentiation, apoptosis, and extracellular matrix components may provide new ideas for the repair of Cd-induced cartilage damage and the treatment of articular cartilage diseases. It is best to observe the effect of MMP-9 on collagen expression by MMP-9 inhibitor.

**Figure 8:**
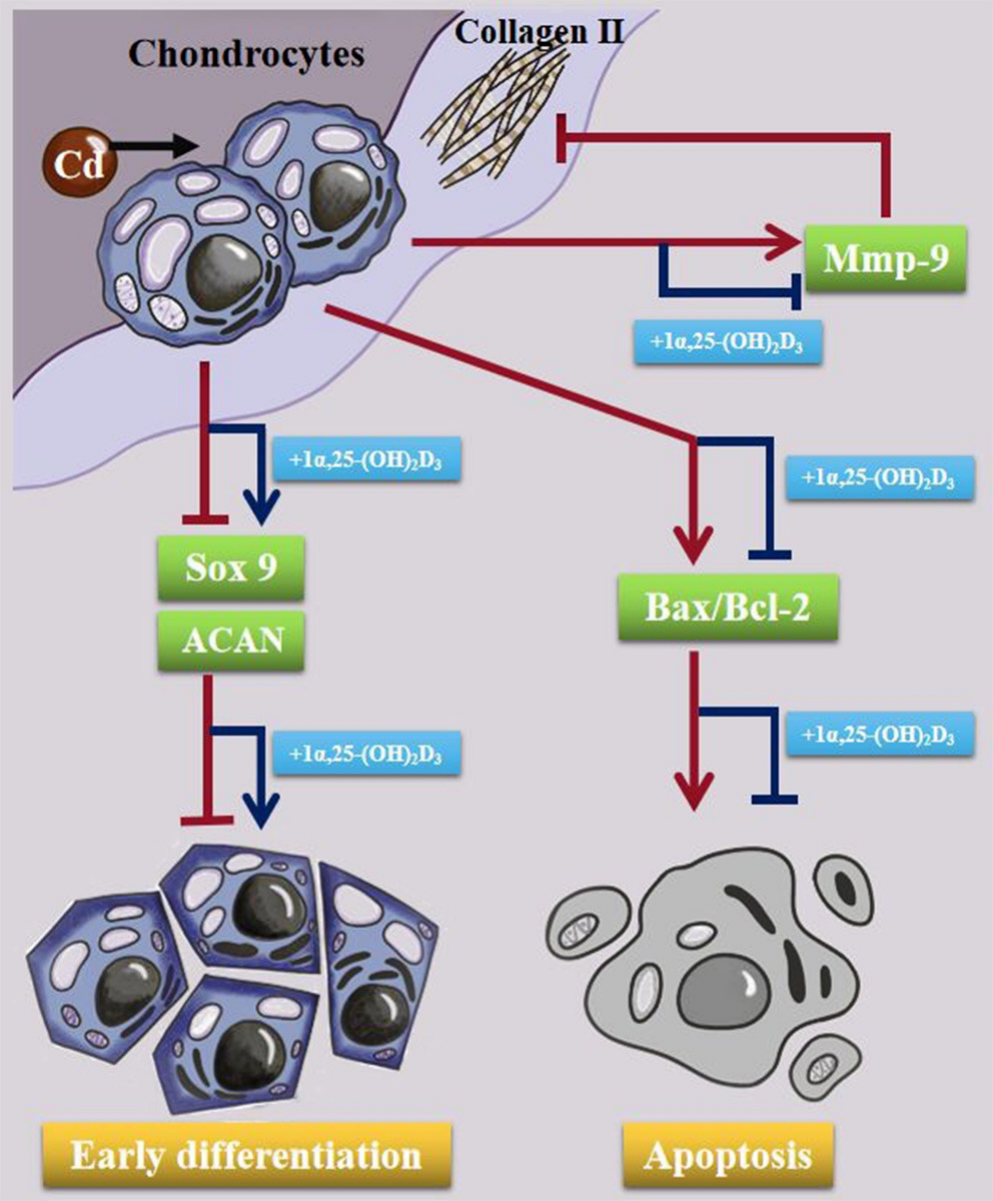
Schematic diagram showing the effect of Cd in chondrocytes. Cd inhibited the expression of *Sox9* and *ACAN* mRNA, eventual inhibition early differentiation in chondrocytes. Cd upregulated Bax/Bcl-2 expression and induced apoptosis in chondrocytes. Cd also promoted MMP-9 expression and COL2A1 degradation in the cells. 10^−8^ M 1α,25-(OH)_2_D_3_ alleviated the toxic damage of Cd to chondrocytes.

In summary (Figure 8), Cd inhibited chicken chondrocytes differentiation by inhibiting chondrocyte differentiation markers, Sox9 and ACAN mRNA. It also up-regulated Bax/bcl-2 ratio and induced chicken chondrocyte apoptosis. Furthermore, Cd inhibited extracellular matrix, such as collagen type IIα1 (COL2A1) expression *via* up-regulating matrix metalloproteinase-9 (MMP-9) in chicken chondrocytes. Application of 1α,25-(OH)_2_D_3_ before Cd treatment can relieve Cd toxicity in chicken chondrocytes.

## Data Availability Statement

The raw data supporting the conclusions of this article will be made available by the authors, without undue reservation.

## Author Contributions

JG, SL, and ZL designed this project. SL, GW, and XZ performed the experiments and analyzed the results. YY, XL, and JB revised the manuscript. JG and SL drafted this manuscript. All authors revised final version of the manuscript.

## Conflict of Interest

The authors declare that the research was conducted in the absence of any commercial or financial relationships that could be construed as a potential conflict of interest.

## Abbreviations

1α, 25-(OH)_2_D_3_, 1α: 25-dihydroxyvitamin D_3_
ACAN: aggrecan
ALP: alkaline phosphatase
Cd: cadmium
COL2A1: collagen type IIα1
GAGs: glycosaminoglycans
GAPDH: glyceraldehyde 3-phosphate dehydrogenase
MMP-9: matrix metalloproteinase-9
OA: osteoarthritis
Sox9: sex determining region Y box 9.
